# Depletion of FAP^+^ cells reduces immunosuppressive cells and improves metabolism and functions CD8^+^T cells within tumors

**DOI:** 10.18632/oncotarget.7818

**Published:** 2016-03-01

**Authors:** Ying Zhang, Hildegund C.J. Ertl

**Affiliations:** ^1^ Gene Therapy and Vaccines Program, University of Pennsylvania, Philadelphia, Pennsylvania, USA; ^2^ The Wistar Institute, Philadelphia, Pennsylvania, USA

**Keywords:** FAP^+^ fibroblasts, cancer vaccine, metabolic stress, T cell exhaustion, immunosuppressive cells

## Abstract

The tumor stroma, which is essential to support growth and metastasis of malignant cells, provides targets for active immunotherapy of cancer. Previous studies have shown that depleting fibroblast activation protein (FAP)-expressing stromal cells reduces tumor progression and concomitantly increases tumor antigen (TA)-specific T cell responses. However the underlying pathways remain ill defined. Here we identify that immunosuppressive cells (ISCs) from tumor-bearing mice impose metabolic stress on CD8^+^T cells, which is associated with increased expression of the co-inhibitor PD-1. In two mouse melanoma models, depleting FAP^+^ stroma cells from the tumor microenvironment (TME) upon vaccination with an adenoviral-vector reduces frequencies and functions of ISCs. This is associated with changes in the cytokine/chemokine milieu in the TME and decreased activity of STAT6 signaling within ISCs. Decreases in ISCs upon FAP^+^stromal cell depletion is associated with reduced metabolic stress of vaccine-induced tumor infiltrating CD8^+^T cells and their delayed progression towards functional exhaustion, resulting in prolonged survival of tumor-bearing mice.

## INTRODUCTION

Solid tumors are composed of neoplastic cells and tumor stroma. The stroma, which includes connective tissue, cancer-associated fibroblasts (CAFs), blood vessels and infiltrating inflammatory cells, is essential for progression of solid tumors [1-4]. Tumor stroma also protects malignant tumor cells from an onslaught by the immune system through subverting protective immune responses, while supporting those that are immunosuppressive [5-7]. Targeting tumor stroma is thus being explored for treatment of cancer patients [8-10].

The supporting stroma of melanoma contains an abundance of CAFs, which are functionally distinct from fibroblasts in normal tissues [11-13]. One key distinguishing feature is their selective expression of FAP, which is not present at high levels on cells of a healthy adult organism [14-16]. FAP^+^ stromal fibroblasts are essential to maintain the TME and promote cancer progression. They inhibit TA-specific immune responses by producing cytokines and chemokines, which attract immunosuppressive cells (ISCs) including regulatory T cells (Tregs), myeloid-derived suppressor cells (MDSCs) and tumor-associated macrophages (TAMs) [17, 18]. Factors secreted by FAP^+^ stromal cells may also interfere with T cell-tumor cell interactions and hinder tumor cell lysis [19]. Genetic depletion of FAP, vaccines targeting FAP or T cells with a FAP-specific chimeric antigen receptor (CAR) inhibit tumor growth in part by enhancing tumor-specific immune responses [20-23]; however the underlying mechanisms remain poorly understood.

T cell responses within tumors are impaired by numerous mechanisms such as increases of co-inhibitors upon chronic antigen stimulation and accumulation of tumor-infiltrating ISCs. Recently it has been reported that lack of glucose within the TME poses metabolic stress on tumor-infiltrating T lymphocytes (TILs), which contributes to their functional exhaustion and impairs their antitumor performances [24, 25]. We hypothesized that depletion of FAP^+^ stromal cells may reduce the metabolic stress of TA-specific TILs and thereby improve their effector functions and the overall efficacy of active immunotherapy. To test this hypothesis, we used a replication-defective adenovirus (Ad)-based vaccine expressing FAP given together with an Ad vaccine expressing multiple epitopes from melanoma-associated antigens (MAAs) in two mouse melanoma models. Our data show that vaccination against FAP significantly improves the therapeutic efficacy of the traditional cancer vaccine by destroying FAP^+^ stroma cells. In addition it reduces numbers and functions of tumor-infiltrating ISCs by changing the cytokine/chemokine milieu within the TME and inhibiting the activity of the STAT6 signaling pathway within ISCs. We show *in vitro* that ISCs enhance the mitochondrial metabolic stress of activated CD8^+^T cells and increase expression of the co-inhibitor PD-1. In the same token, the decreased levels of ISCs within the TME upon FAP vaccination is associated with reduced metabolic stress of vaccine-induced MAA-specific CD8^+^T cells, improved frequencies and effector functions of these cells and their delayed progression towards exhaustion.

Our data support further exploring the tumor-stroma-targeting vaccines for active immunotherapy of cancer.

## RESULTS

### The AdC68-mFAP vaccine elicits robust antibody and T cell responses in different mouse melanoma models

**Figure 1:**
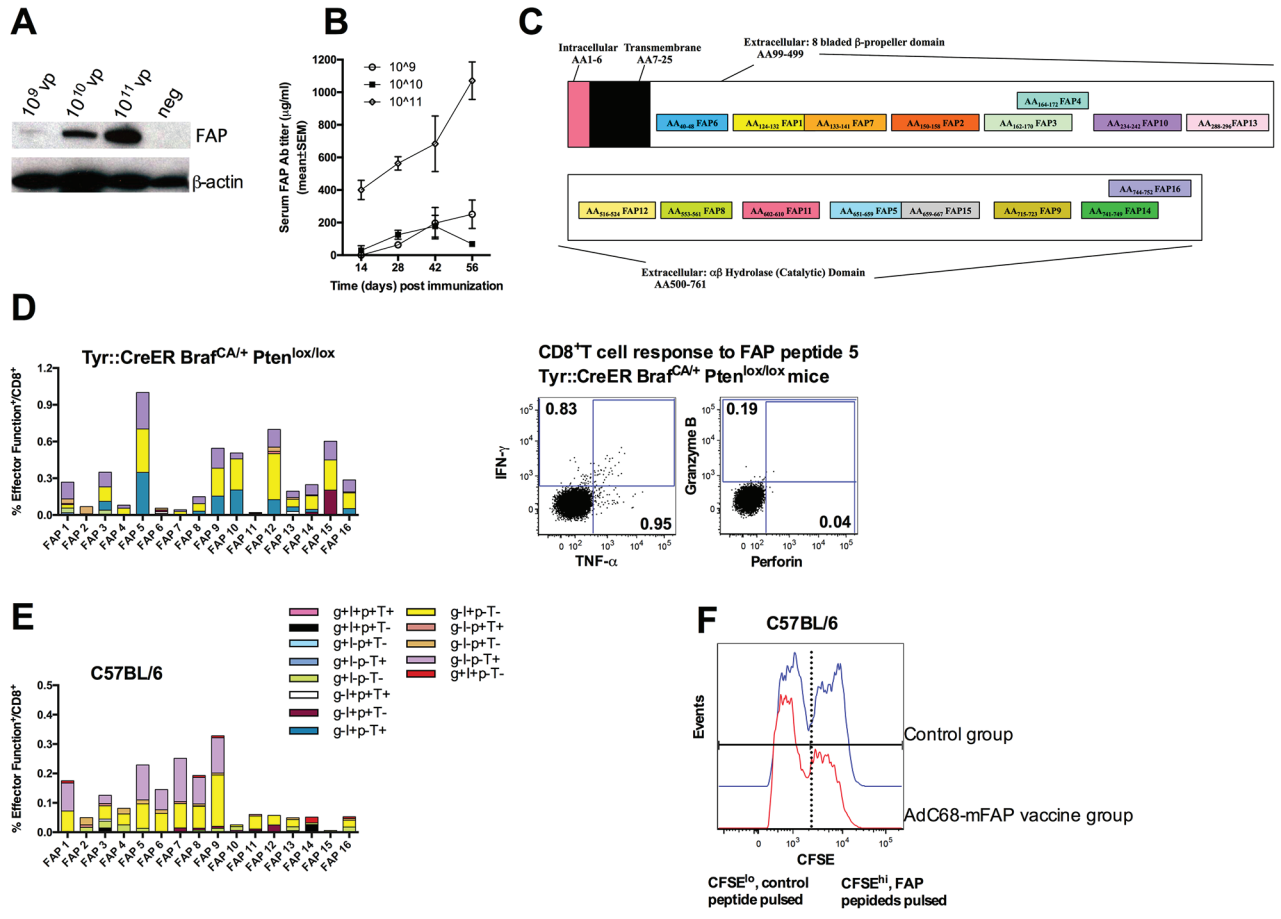
The AdC68-mFAP vaccine induces FAP-specific antibody and CD8^+^T cell responses **A.** HEK 293 cells were infected with different doses of AdC68-mFAP vector and protein was harvested 48 hours later. Full-length murine FAP was visualized by Western blot using β-actin as an internal control. **B.** FAP-specific antibody responses elicited by the AdC68-mFAP vaccine at different time points after vaccination. Results show mean values of FAP antibody titers in serum with standard error of mean (SEM) determined by indirect ELISA. **C.** Schematic cartoon shows different components of FAP and the 16 CD8^+^T cell epitopes within FAP that are predicted to bind H-2K^b^ or H-2D^b^ with high affinity. **D.** Left: Magnitude and polyfunctions of CD8^+^T cells directed to individual FAP epitopes in transgenic mice. Right: Representative flow plots illustrate vaccine-induced CD8^+^T cell response to FAP peptide 5. The production of IFN-γ, TNF-α, granzyme B and perforin were measured. **E.** Magnitude and polyfunctions of CD8^+^T cell responses to individual FAP epitopes in C57BL/6 mice. (D-E) Color scheme illustrates different combinations of factors that were produced. **F.** Representative histograms show *in vivo* cell lysis by AdC68-mFAP vaccine-induced CD8^+^T cells of CFSE^hi^ cells pulsed with FAP peptides. Blue histograms: CFSE^+^ splenocytes from mice vaccinated with control vector 2 weeks earlier. Red histogram: CFSE^+^ splenocytes from mice vaccinated with AdC68-mFAP vector 2 weeks earlier.

To achieve immune-mediated destruction of the tumor stroma, we developed a vaccine based on a replication-defective Ad vector of chimpanzee serotype 68 (AdC68), which expresses full-length murine FAP protein from a CMV-promoter driven transgene incorporated into the vector's deleted E1 domain. The vaccine expressed FAP in transduced HEK 293 cells in a dose-dependent fashion (Figure 1A). The vaccine, termed AdC68-mFAP, elicited robust FAP-specific antibody responses in mice as tested by a FAP-specific ELISA with sera from individual vaccinated mice (Figure 1B). We further tested AdC68-mFAP for induction of FAP-specific CD8^+^T cells by measuring vaccine-induced responses to 16 potential CD8^+^T cell epitopes of mouse FAP (Figure 1C). The epitopes were selected based on their predicted high affinity to MHC class I antigens H-2D^b^ and H-2K^b^. The vaccine was tested in wild-type C57BL/6 mice and transgenic Tyr::CreER, Braf^CA/+^Pten^lox+/lox+^mice. The transgenic mice were genetically engineered to develop melanoma upon Cre-mediated disruption of Pten expression [26]. This model, which recapitulates the genetic mutations of human melanoma, is a highly clinically relevant model for pre-clinical evaluation of therapies for melanoma. In both mouse strains AdC68-mFAP induced CD8^+^T cells produced mainly interferon (IFN)-γ or tumor necrosis factor (TNF)-α in response to *in vitro* stimulation with FAP-derived peptides representing each of the 16 epitopes expressed by the vaccine (Figure 1D, 1E). Frequencies of FAP-specific CD8^+^T cell responses were significantly higher in transgenic mice. FAP-specific CD8^+^T cells elicited in C57BL/6 mice mainly recognized epitopes 1 and 5-9, while those in Braf^CA/+^Pten^lox+/lox^ mice mainly responded to epitopes 5, 9, 10, 12 and 15. To confirm that the FAP-specific CD8^+^T cells were able to kill their target cells, we performed *in vivo* cytotoxicity assay in C57BL/6 mice immunized with AdC68-mFAP or a control Ad vector. Syngeneic splenocytes were pulsed either with FAP peptides (i.e., peptides 1, 5, 7, 8 and 9) or a control peptide. They were then labeled with high or low concentrations of CFSE, respectively. The two cell populations were mixed in a 1:1 ratio and transferred to recipient mice that had been immunized 2 weeks earlier with either AdC68-mFAP or a control Ad vector. Compared to control mice, the transferred cells showed significant loss of the CFSE^hi^ FAP peptides-pulsed cell population in relation to the CFSE^low^ control population in AdC68-mFAP vaccinated mice (34.5% of CFSE^hi^ cells were lysed in the AdC68-mFAP vaccine group, FAP group vs. control group p=0.0011), suggesting that FAP-specific CD8^+^T cells elicited by AdC68-mFAP vaccine mediated specific target cell lysis (Figure 1F). Together these data show that the AdC68-mFAP vaccine is immunogenic and induces robust FAP-specific B and T cell responses in different mouse strains.

### AdC68-mFAP delays tumor growth and improves survival of melanoma-bearing mice

**Figure 2:**
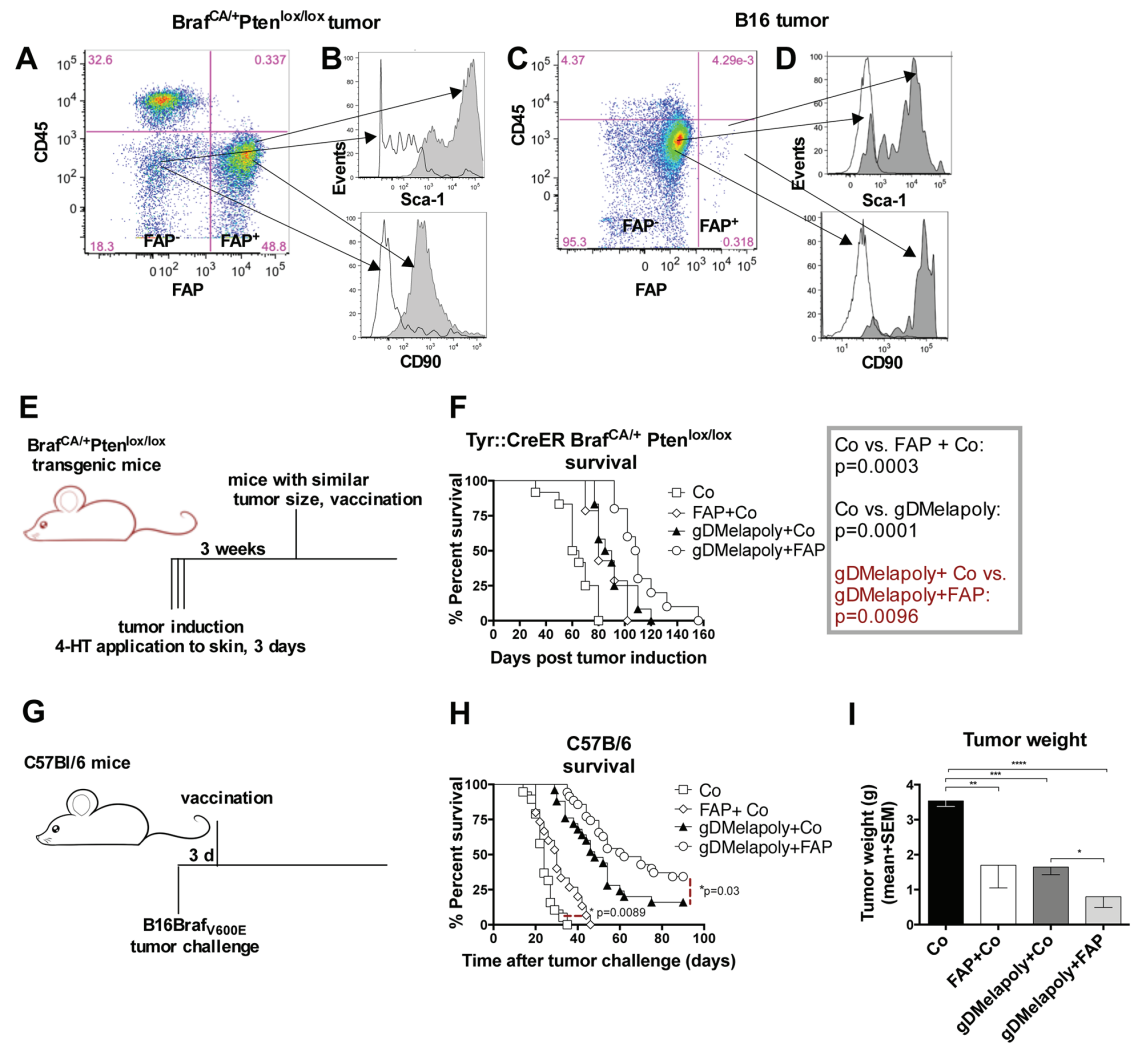
Vaccination with the AdC68-mFAP vector improves survival of tumor-bearing mice **A, C.** Representative flow plot shows the presence of CD45^−^FAP^+^cells within tumors from Braf^CA/+^Pten^lox/lox^ transgenic mice (A) or within B16 tumors (C). **B, D.** Histograms indicate the expression of mesenchymal stroma cell markers CD90 and Sca-1 on CD45^−^FAP^+^ (dark grey) and CD45^−^FAP^−^ cells (white) in tumors from transgenic mice (B) or in B16 tumors (D). **E.** Schematic representation of experimental set up to test vaccine efficacy in transgenic mice. Tumors were induced in transgenic mice by 4-HT treatment for 3 consecutive days. Mice were vaccinated with different vectors 3 weeks after tumor induction. **F.** Graph shows Kaplan-Meier survival curves of mice that received the different vaccine regimens (n=10-14/group). Open square: control group (AdC68-gD vector); Diamond: control+AdC68-mFAP vaccine group; Black triangle: AdC68-gDMelapoly+ AdC68-gD vaccine group; Circle: AdC68-gDMelapoly+ AdC68-mFAP vaccine group. AdC68-gD vs. FAP+ AdC68-gD: p=0.0003; AdC68-gD vs. gDMelapoly+control: p=0.0001; gDMelapoly+ AdC68-gD vs. gDMelapoly+FAP: p=0.0096. **G.** Schematic representation of experimental set up to test vaccine efficacy in mice bearing transplantable B16 tumors. C57BL/6 mice were challenged with B16 tumor cells and vaccinated three days later with different vectors. **H.** Graph shows Kaplan-Meier survival curves of mice that received the different vaccine regimens (n=15-35/group). Symbols representing each vaccine group are the same as those used in 2F. Control vs. FAP+control: p=0.0089; gDMelapoly+ AdC68-gD vs. gDMelapoly+FAP: p=0.03. **I.** Tumor weight comparisons among different vaccine groups on day 25 after B16 tumor challenge. Data are presented as mean with SEM. AdC68-gD vs. FAP+ AdC68-gD: p=0.014; AdC68-gD vs. gDMelapoly+AdC68-gD: p=0.0001; AdC68-gD vs. gDMelapoly+FAP: p<0.0001; gDMelapoly+AdC68-gD vs. gDMelapoly+FAP: p=0.03.

To assess if the FAP vaccine was likely to influence tumor progression, we analyzed FAP^+^ tumor stroma cells from Braf^CA/+^Pten^lox+/lox+^mice that upon treatment with 4-hydroxyltamoxifen (4-HT) developed tumors. C57Bl/6 mice challenged with a B16F10 cell line modified to express Braf_V600E_ (B16Braf_V600E_, referred to as B16) were tested as well. The Braf_V600E_ epitope, which is highly prevalent in human melanoma, was included to better assess the potential of the vaccine in treating melanoma patients. Expression of FAP within the stroma of ~ 4 week-old Braf^CA/+^Pten^lox+/lox+^ and B16 tumors was confirmed at the mRNA (not shown) and protein level (Figure 2A, 2C). A high percentage (~40-50%) of CD3^−^CD14^−^CD45^low^ cells from tumors of the transgenic mice stained positive for FAP. In contrast, proportion of FAP^+^ cells was lower in B16 tumors. The expression of mutated Braf within the B16 tumor cells may have affected levels of FAP^+^ cells, as a different B16.F10 tumor with wild-type Braf but modified to express GFP had markedly higher percentages of FAP^+^ cells (~ 25%, data not shown). Most of the FAP^+^ cells within either tumor only expressed low to intermediate levels of CD45. Compared to FAP^−^ cells, FAP^+^ cells from either tumors expressed significantly higher levels of mesenchymal stromal cell markers CD90 and Sca-1, confirming the stromal cell lineage of FAP^+^ cells [14] (Figure 2B, 2D).

To measure the effect of AdC68-mFAP vaccination on tumor progression, we first used Braf^CA/+^Pten^lox+/lox+^ mice, in which tumors had been initiated 3 weeks earlier (Figure 2E). We vaccinated mice bearing similar sized tumors with either a control AdC68 vector or AdC68-mFAP. Additional groups received AdC68-mFAP together with a tumor-cell targeting melanoma vaccine termed AdC68-gDMelapoly. AdC68-gDMelapoly vaccine expresses a series of melanoma-associated antigen (MAA) epitopes within herpes simplex virus glycoprotein D (gD) and can elicit robust CD8^+^T cell responses to multiple MAAs as described before [27]. Other mice received AdC68-gDMelapoly mixed with a control AdC68 vector, the latter to ensure that mice received equal doses of vaccine. AdC68-mFAP or AdC68-gDMelapoly vector given alone each achieved significant delay in tumor progression compared to the control vaccine. Tumor growth was comparable in the two groups that received single vectors (p=0.31) (Figure 2F), but was further retarded when the vaccines were combined (gDMelapoly+Co vs. gDMelapoly+FAP: p=0.0096).

We further assessed vaccine efficacy in the transplantable B16 tumor model. C57Bl/6 mice were vaccinated with the different vectors three days after tumor challenge (Figure 2G). We chose an early time point after tumor challenge to assess the vaccines, as our previous studies showed that the AdC68-gDMelapoly vaccine, which completely protects mice that are vaccinated before tumor challenge can only rarely lead to cures if given three days after tumor challenge [27]. Compared to the control group, mice immunized only with AdC68-mFAP showed delayed tumor progression and significantly prolonged survival (p=0.0089, Figure 2H). Vaccine efficacy was further improved when mice were immunized with a mixture of AdC68-mFAP and AdC68-gDMelapoly; while ~16% of AdC68-gDMelapoly-vaccinated mice were completed protected from tumor challenge, immunization with the vaccine mixture early after tumor challenge more than doubled the numbers of mice (~35%) that remained tumor-free for at least 90 days after tumor induction. In mice that developed tumors, those that received the AdC68-mFAP or AdC68-gDMelapoly vaccine alone showed significantly reduced tumor weight on day 25 after tumor challenge compared to control mice. Combining AdC68-gDMelapoly with AdC68-mFAP further decreased tumor weight (Figure 2I).

Both sets of data confirm that targeting FAP^+^ tumor stroma cells results in significantly prolonged survival of melanoma-bearing mice. Combining a conventional tumor cell-targeting vaccine with a vaccine directed against the tumor stroma offers further therapeutic benefits in mouse melanoma models.

### AdC68-mFAP-induced CD8^+^T cell responses reduce FAP^+^ stromal cells within the TME

**Figure 3:**
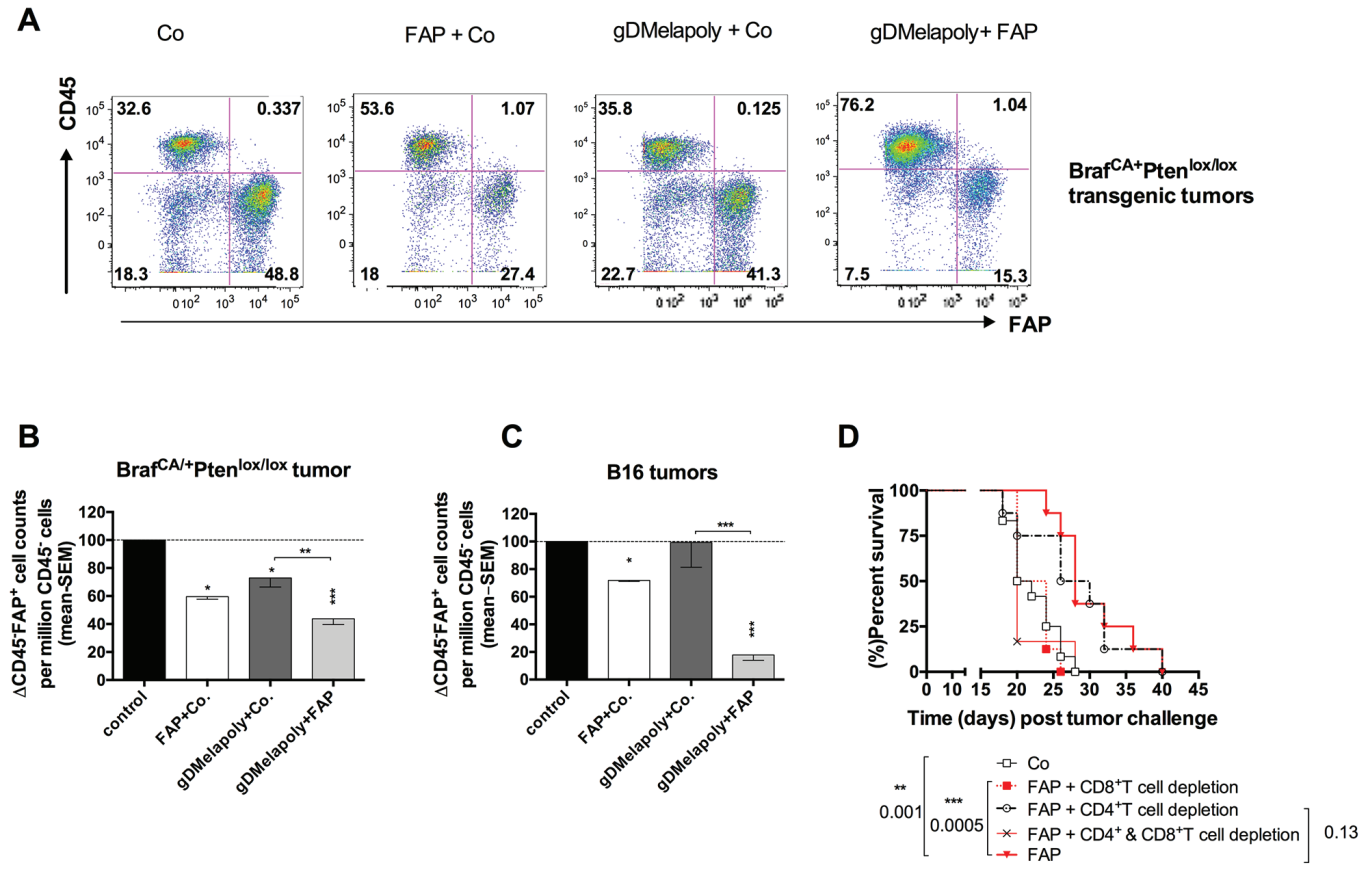
Immunization with AdC68-mFAP reduces numbers of FAP^+^ cells within both transgenic Braf^CA/+^Pten^lox/lox^ and transplantable B16 tumors in a CD8^+^T cell dependent manner **A.** Representative flow plots show percentages of CD45^−^FAP^+^ cells from 3 month-old tumors of transgenic mice that received AdC68-gD (control), AdC68-mFAP+AdC68-gD, AdC68-gDMelapoly+AdC68-gD or AdC68-gDMelapoly+AdC68-mFAP 3 weeks after initial tumor-induction with 4-HT. **B.** Relative numbers of CD45-FAP^+^ cells per million CD45^−^CD3^−^CD14^−^CD19^−^ cells from 3 month-old tumors isolated from transgenic mice that received the control vector (AdC68-gD, black bar), the AdC68-mFAP+AdC68-gD (open bar), the AdC68-gDMelapoly+ AdC68-gD (dark grey bar), or the AdC68-gDMelapoly+AdC68-FAP (light grey bar) (n=4-5/group). Data were normalized to results from control group. AdC68-gD vs. FAP+ control: p=0.015; AdC68-gD vs. gDMelapoly+FAP: p=0.0006; AdC68-gD vs. gDMelapoly+AdC68-gD: p=0.049; gDMelapoly+AdC68-gD vs. gDMelapoly+FAP: p=0.0083. **C.** Relative numbers of CD45-FAP^+^ cells per million CD45^−^CD3^−^CD14^−^CD19^−^ cells from 1-month B16 tumors isolated from C57BL/6 mice that received different combinations of vectors 3 days after tumor challenge (n=5-10/group). Data were normalized to results from the control group. AdC68-gD vs. FAP+AdC68-gD: p<0.0001; AdC68-gD vs. gDMelapoly+FAP: p<0.00001; AdC68-gD vs. gDMelapol+AdC68-gD: p=0.9625; gDMelapoly+AdC68-gD vs. gDMelapoly+ FAP: p=0.0001. FAP+AdC68-gD vs. gDMelapoly+FAP: p=0.0001. **D.** Kaplan-Meier survival curves of mice challenged with tumor cells and vaccinated with control or AdC68-mFAP vector. Mice were depleted of CD8^+^, CD4^+^ T cells or both (n=8-12/group). Statistical significant differences in terms of survival length and corresponding p-values are marked on the graph.

To determine the mechanism by which the AdC68-mFAP vaccine delayed tumor progression, we first analyzed whether AdC68-mFAP vaccine-induced immune responses could destroy FAP^+^ stroma cells. Tyr::CreER, Braf^CA/+^Pten^lox+/lox+^ transgenic mice bearing 3 week-old tumors and C57Bl/6 mice with 3 day-old B16Braf_V600E_ tumors were vaccinated with the control vector only, AdC68-mFAP with the control vector or AdC68-gDMelapoly mixed with either the control vector or the AdC68-mFAP vector. Numbers and percentages of CD45^−^FAP^+^ stroma cells within tumors were measured 3 month later from transgenic mice and ~4 weeks later from B16 tumor-bearing mice. AdC68-mFAP given alone or with AdC68-gDMelapoly significantly reduced FAP^+^ stroma cells within tumors in both models, reflected by decreases in percentages (Figure 3A) and numbers (Figure 3B, 3C). Within transgenic tumors the FAP vaccine caused a significant reduction in FAP^+^ cells and this was slightly more pronounced if the AdC68-gDMelapoly vaccine was given simultaneously (Figure 3B). Mice immunized only with AdC68-gDMelapoly also had lower levels of FAP^+^ cells. We assume that this may reflect that FAP^+^ cells become more frequent during tumor growth so that a vaccine that delays tumor progression also reduces accumulation of FAP^+^ cells. Results differed for B16 tumors. In this model the FAP vaccine reduced FAP^+^ cells as in the transgenic tumors. In contrast, the AdC68-gDMelapoly vaccine given together with a control vector did not affect levels of the tumors' FAP^+^ stroma cells (Figure 3C). Nevertheless, when AdC68-gDMelapoly and AdC68-mFAP vectors were combined FAP depletion was more pronounced than upon vaccination with AdC68-mFAP only.

Reduction of FAP^+^ cells upon vaccination with AdC68-mFAP suggests their depletion by vaccine-induced T cells. This was confirmed indirectly by testing whether T cells are required for AdC68-mFAP-mediated delay in tumor progression. We depleted CD4^+^ or CD8^+^ or both T cell subsets from mice challenged with B16 tumors and then vaccinated them 3 days later with AdC68-mFAP. Depletion of CD8^+^T cells completely abrogated the effect of the AdC68-mFAP vaccine on tumor progression. Depletion of CD4^+^T cells had no effect (Figure 3D).

### ISCs that intensify metabolic stress of *in vitro* activated CD8^+^T cells are reduced upon FAP^+^ stroma cell depletion

**Figure 4:**
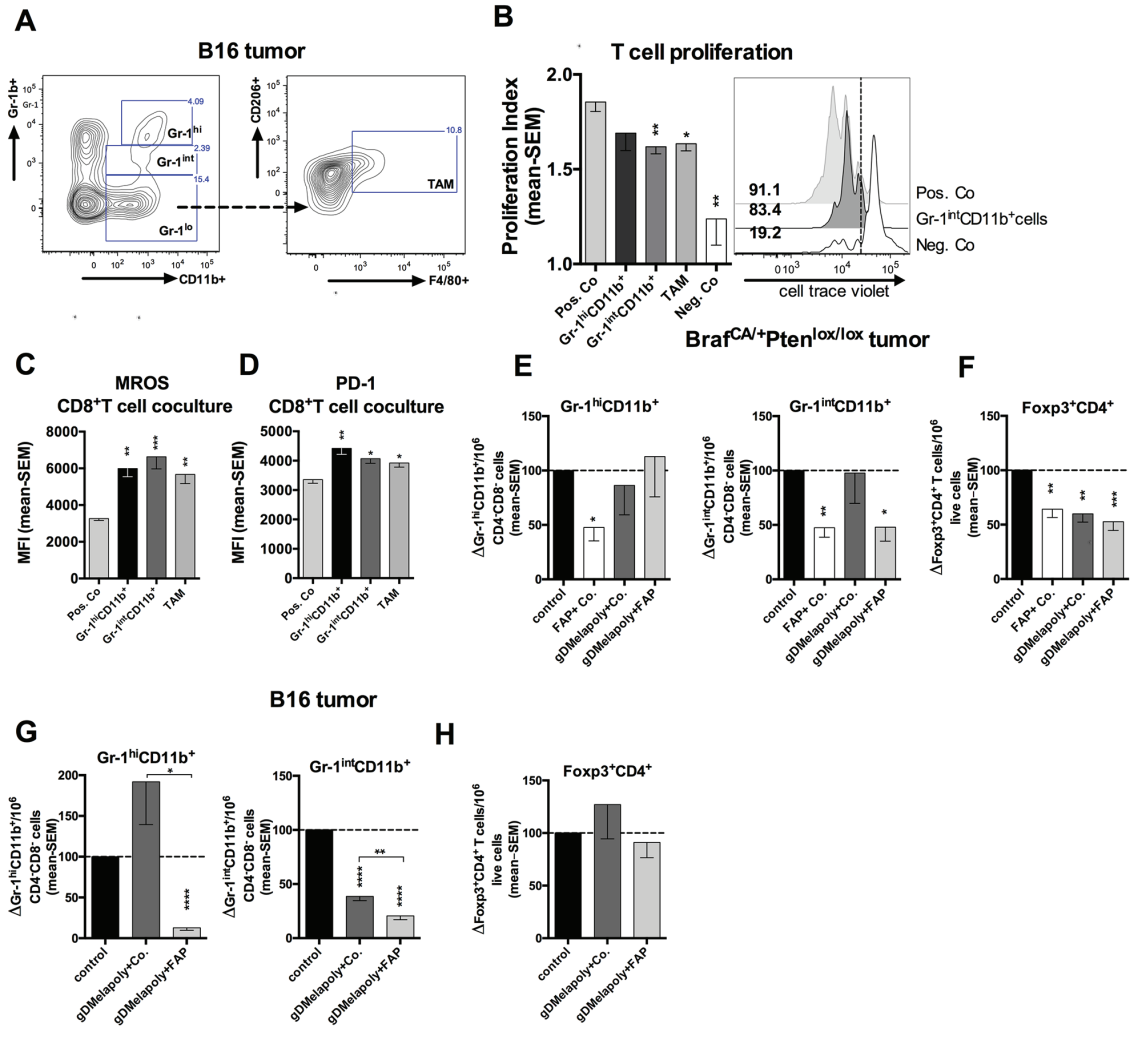
ISCs that enhance the metabolic stress and PD-1 expression of activated CD8^+^T cells were reduced by FAP^+^ tumor stromal cell depletion **A.** Gating strategy for Gr-1^hi^ and Gr-1^int^ MDSCs and CD206^+^F4/80^+^ M2 type TAMs. **B.** Gr-1^int^CD11b^+^ MDSCs and TAMs inhibit proliferation of CD8^+^T cells *in vitro*. Enriched CD8^+^T cells were labeled with celltrace violet and stimulated with anti-CD3 and anti-CD28 for 4 days, Gr-1^int^CD11b^+^ MDSCs were added to T cells at a ratio of 1:5 from day 0. Activated CD8^+^T cells without ISCs were used as positive control while CD8^+^T cells cultured without antibody stimulation were used as a negative control. Left: Proliferation index of CD8^+^T cells with or without stimulation or cocultured with MDSCs or TAM (n=4/group). Positive control vs. Gr-1^int^CD11b^+^co-culture: p=0.0023; positive control vs. TAM co-culture: p=0.023; positive control vs. negative control: p=0.009. Right: Histograms show cell proliferation of representative samples. Numbers on the left of the histograms show percentages of cells with reduced celltrace violet levels. **C.** Enriched CD8^+^T cells from spleens of naive mice were stimulated *in vitro* with or without different subsets of ICSs from tumor-bearing mice. MROS levels in CD8^+^T cells stimulated for 5 days under different culture conditions are shown as mean MFI values with SEM. Positive Co. vs. Gr-1^hi^: p=0.0018; vs. Gr-1^int^: p=0.0003; vs. TAM: p=0.0047. **D.** PD-1 expression on CD8^+^T cell on day 5 after co-culture with different ISC subsets are shown as MFI with SEM. Positive Co. vs. Gr-1^hi^: p=0.0022; vs. Gr-1^int^: p=0.028; vs. TAM: p=0.029. **E–F.** Transgenic mice bearing 3-week tumors were vaccinated with control vector (AdC68-gD, black bar), FAP+AdC68-gD (empty bar), gDMelapoly+AdC68-gD (dark grey bar) or gDMelapoly+FAP (light grey bar) (n=5/group). Data show normalized cell counts of Gr-1^hi^ and Gr-1^int^MDSCs over CD4^−^CD8^−^ live cells (E) or normalized CD4^+^Foxp3^+^ cells (F) over live cells in 3 month-old tumors of mice from the different vaccine groups. **G–H.** Mice bearing 3 day-old B16 tumors were vaccinated with control vector (AdC68-gD, black bar), gDMelapoly+AdC68-gD (dark grey bar) or gDMelapoly+FAP (light grey bar) (n=14-15/group). Data show normalized cell counts of Gr-1^hi^ and Gr-1^int^MDSCs over CD4^−^CD8^−^ live cells (G) or normalized CD4^+^Foxp3^+^ cells (H) over live cells in 1 month-old tumors of mice from the different vaccine groups. *: p<=0.05; **: p<0.01; ***: p<0.001; ****: p<0.0001.

Cytokines and chemokines produced within tumors are known to recruit ISCs, such as MDSCs and FoxP3^+^CD4^+^ regulatory T cells (Treg) [28]. We hypothesized these ISCs may enhance the metabolic stress of MAA-specific TILs within TME and contributes to their functional exhaustion. We measured two subsets of MDSCs, i.e., monocytic (MO) MDSCs, which are phenotypically Gr-1^int^CD11b^+^, and granulocytic (polymorphonuclear, PMN) MDSCs, which are Gr-1^hi^CD11b^+^ and TAMs (Figure 4A). Most of TAMs within TME were skewed towards a M2 phenotype with high expression of mannose receptor CD206 [29]. We confirmed *in vitro* that the ISCs affected T cell proliferation by activating splenic naïve CD8^+^T cells *in vitro* with antibodies to CD3 and CD28. Proliferation tested for at 5 days after activation significantly decreased upon co-culture with MO-MDSCs and TAMs. Some inhibition was seen upon co-culture with PMN-MDSCs although this failed to reach significance (Figure 4B).

To determine whether ISCs affect T cell metabolism, we stimulated CD8^+^T cells from spleens of naïve mice *in vitro* in presence of different populations of ISCs isolated from B16 tumor-bearing mice. Levels of mitochondrial reactive oxygen species (MROS) within activated CD8^+^T cells were measured on day 5 of culture. T cells upon activation increasingly use glycolysis for production of energy while resting T cells primarily use the more efficient tricarboxylic acid (TCA) cycle and oxidative phosphorylation (OXPHOS) [30]. MROS, which is mainly produced by OXPHOS, is highly toxic and can induce cell damage and death through activating intracellular signaling pathways [31]. In healthy cells MROS is rapidly converted to water and oxygen. Its accumulation within cells is a hallmark of metabolic stress indicative of mitochondrial dysfunctions. Activated CD8^+^T cells co-cultured with MDSCs and TAMs showed significantly higher MROS levels compared to those stimulated without ISCs (Figure 4C), suggesting that ISCs impose metabolic stress on activated CD8^+^T cells. We next assessed whether increased metabolic stress contributes to inhibitory signaling in activated CD8^+^T cells by measuring expression of the co-inhibitor PD-1. PD-1 initially increases on activated CD8^+^T cells upon T cell receptor and CD28 ligation [32]. Its constitutive high expression is viewed as a hallmark of exhaustion that limits the effectiveness of CD8^+^TILs [33]. PD-1 dampens T cell responses in part by inhibiting the Akt/mTOR pathway and thereby energy production through glycolysis [34]. In our CD8^+^T cells-ISCs co-culture system, addition of ISCs significantly increased PD-1 expression on CD8^+^T cells (Figure 4D).

We next analyzed whether depleting FAP^+^ stroma cells can reduce ISCs within the TME. Transgenic mice bearing 3 months old tumors of similar sizes were analyzed first. Tumors from mice that received AdC68-mFAP alone compared to those from the control group showed significantly reduced percentages of both MDSC subsets; this was not achieved in mice that received AdC68-gDMelapoly with a control vector (Figure 4E). Combining AdC68-mFAP with AdC68-gDMelapoly caused a reduction in the more suppressive Gr-1^int^CD11b^+^MDSC subset while percentages of Gr-1^hi^CD11b^+^MDCSs reverted back to levels seen in control mice. Numbers of Tregs declined upon immunization with either vaccine regimen (Figure 4F), indicating that this was unrelated to FAP but more likely reflected the effect of enhanced immune response within the tumor upon vaccination with Ad vectors.

In B16 tumors we excluded the group of mice immunized with AdC68-mFAP only, as in this model reduction of FAP^+^ cells was significantly more pronounced by a vaccine regimen that combines AdC68-mFAP with the gDMelapoly vector (Figure 3C). AdC68-gDMelapoly vaccinated-mice had slightly enhanced levels of Gr-1^hi^CD11b^+^ MDSCs; addition of the FAP vaccine reduced this population (Figure 4G). Relative percentages of Gr-1^int^CD11b^+^ MDSCs were reduced upon vaccination with AdC68-gDMelapoly. This reduction became more pronounced in tumors of mice that also received the FAP vaccine, suggesting that depleting FAP^+^ stromal cells contributed to the lower MDSC levels. The vaccines did not reduce Tregs (Figure 4H). In both tumor models frequencies of TAMs were not affected by the FAP vaccine. The vaccines thus had distinct effects on ISC numbers in the two tumor models. Overall these data indicate that in either melanoma model targeting FAP^+^ tumor stroma cells reduces the content of MDSCs within tumors, which may create a more supportive niche for antigen-specific TILs by reducing their metabolic stress.

### Reduced suppressive functions of ISCs upon FAP^+^ stromal cell depletion is linked to changes in JAK-STAT pathway activation

**Figure 5:**
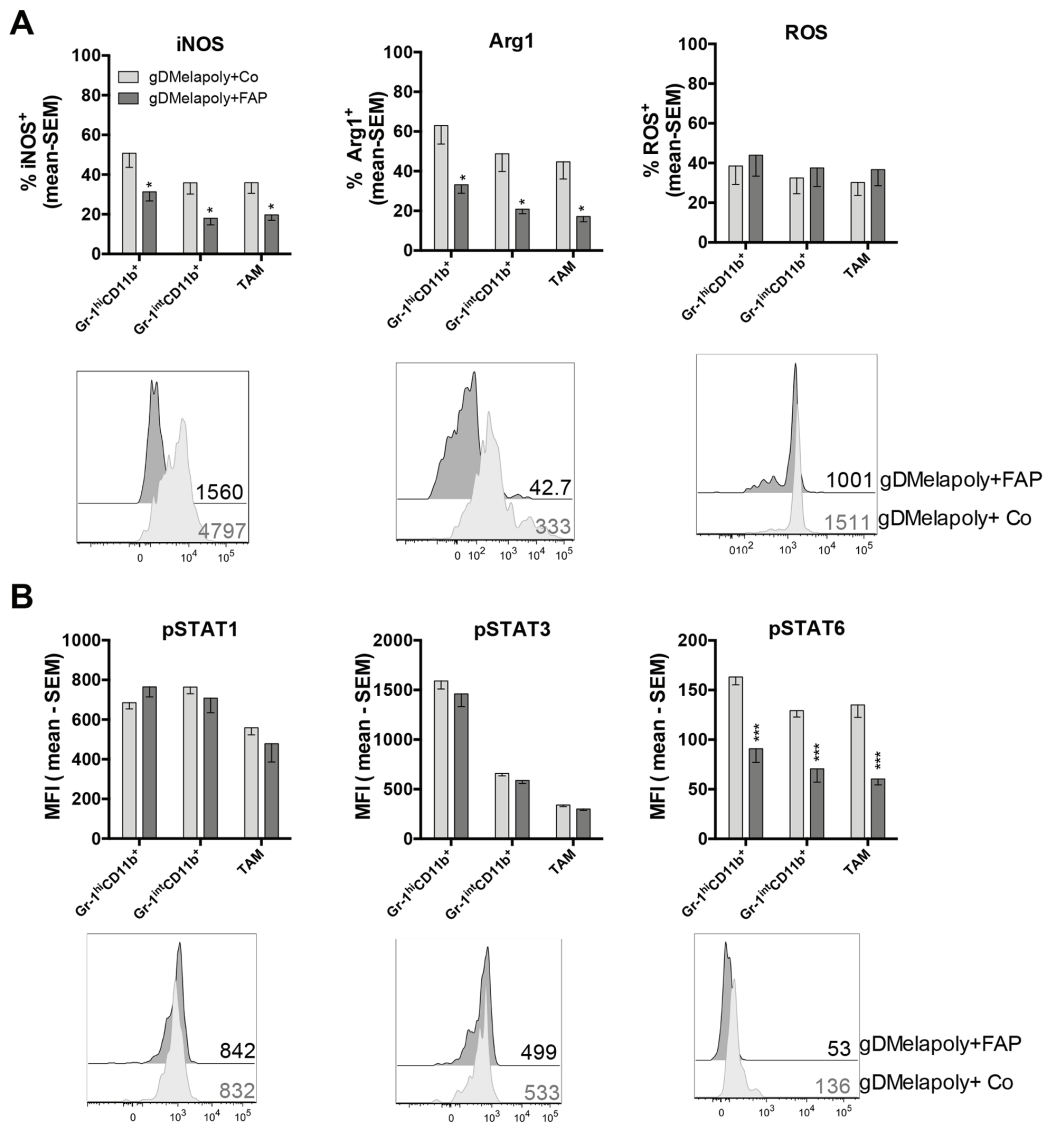
Depleting FAP^+^ stromal cells reduces suppressive functions of ISCs in tumors and decreases their STAT6 activity **A.** Production of iNOS, Arg1 and ROS by MDSCs and TAMs from similar-sized 1 month-old B16 tumors were compared between mice that received AdC68-gDMelapoly+AdC68-gD (light grey bar) or AdC68-gDMelapoly+ AdC68-mFAP (dark grey bar) (n=15/group). Data are presented as percentages of cells positive for iNOS, Arg1 or ROS expression. Lower panel: representative histograms of iNOS, Arg1 and ROS expression in Gr-1^int^ PMN-MDSCs in the two different vaccine groups. Numbers next to histograms indicate MFI values for the factors in the selected samples. **B.** The phosphorylation of transcription factors STAT1, STAT3 and STAT6 were measured in MDSCs and TAMs using the same tumor samples as described in 5A (n=8-10/group). Data are shown as mean MFI values with SEM. Lower panel: histograms show representative pSTAT1/pSTAT3/pSTAT6 expression in Gr-1^int^CD11b^+^MDSC samples from the two vaccine groups. MFI values of pSTAT expression in the selected samples are shown next to the histograms. Statistics shown as * were same as described in Figure 4.

MDSCs and TAMs suppress CD8^+^T cell functions through different mechanisms [28]. It is well-established that they produce high levels of inducible nitric oxide synthase (iNOS) and arginase 1(Arg1), which catabolize and deplete L-arginine, an important amino acid that is required to support T cell proliferation. In addition, iNOS generates nitric oxide (NO), which further inhibits function of T cells. MDSCs produce reactive oxygen species (ROS), which upon reaction with NO form superoxide anion, a metabolite that through nitration of T cell receptors induces T cell unresponsiveness [35]. CAFs secrete chemokine (C-C motif) ligand 2 (CCL2), which recruits MDSCs. Other factors such as granulocytes macrophage colony-stimulating factor (GM-CSF), interleukin (IL)-4, IL-10, IL-13, transforming growth factor (TGF)-β promote differentiation and immunosuppressive functions of MDSCs [28, 36]. We therefore assessed if depleting FAP^+^ CAFs upon vaccination with AdC68-mFAP can change the functional profiles of ISCs. For these experiment we used the B16 model and combined the AdC68-gDMelapoly and AdC68-mFAP vaccines as this regimen as shown in Figure 3C achieved the highest reduction in FAP^+^ cells. Mice bearing 3-day old B16 tumors were vaccinated with AdC68-gDMelapoly together with control or AdC68-mFAP vectors. MO-MDSCs, PMN-MDSCs and TAMs were isolated from similar-sized tumors of the two groups of mice ~4 weeks after vaccination and levels of iNOS, Arg1 and ROS were measured by antibodies staining and flow analysis. Both iNOS and Arg1 expression were significantly decreased within ISCs from tumors of mice that received both MAA- and FAP-targeting vaccines compared to those received only AdC68-gDMelapoly (Figure 5A). ROS levels were comparable between the two vaccine groups for all ICS populations. Collectively, these data suggest that depletion of FAP^+^ cells reduces the immunosuppressive capacities of ISCs by decreasing their ability to produce iNOS and Arg1.

Previous studies showed that factors produced by tumor and tumor stromal cells activate Janus kinase (JAK)-signal transducer and activator of transcription (STAT) pathways in ISCs, which contributes to their expansion and activation [28, 36]. STAT3 is induced by a number of factors including IL-5, IL-6 and IL-10. It is the main transcriptional factor regulating MDSC expansion. STAT1 activated by IFN-γ and STAT6 activated by IL-4 or IL-13 upregulate the expression of iNOS and Arg1 in MDSCs [36]. As vaccination with AdC68-mFAP given together with AdC68-gDMelapoly reduced iNOS and Arg1 expression in MDSCs and TAMs of tumor-bearing mice, we analyzed whether the functional reductions of ISCs were linked to changes in STAT activation. We compared phosphorylated (p)STAT1, STAT3 and STAT6 levels in the three ISCs populations from B16 tumors of mice vaccinated with AdC68-gDMelapoly with either control or AdC68-mFAP vector. Mice received tumors cells and were vaccinated 3 days later; similar-sized tumors from each group were analyzed 4 weeks after vaccination. Addition of the FAP vaccine to AdC68-gDMelapoly had no effect on pSTAT1 or pSTAT3 levels, but significantly decreased pSTAT6 levels in all three ISC subsets (Figure 5B). These data indicate that vaccine-mediated depletion of FAP^+^ cells reduces the suppressive functions of ISCs, which is associated with their decreased activation of the STAT6 pathway.

### Targeting FAP^+^ cells changes cytokine/chemokine production within tumors

Reduced activity of STAT6 in tumor-infiltrating ISCs of mice that received AdC68-mFAP together with AdC68-gDMelapoly most likely reflects vaccine-induced changes in cytokines or chemokines within the TME, which in turn recruit and activate ISCs.

**Figure 6:**
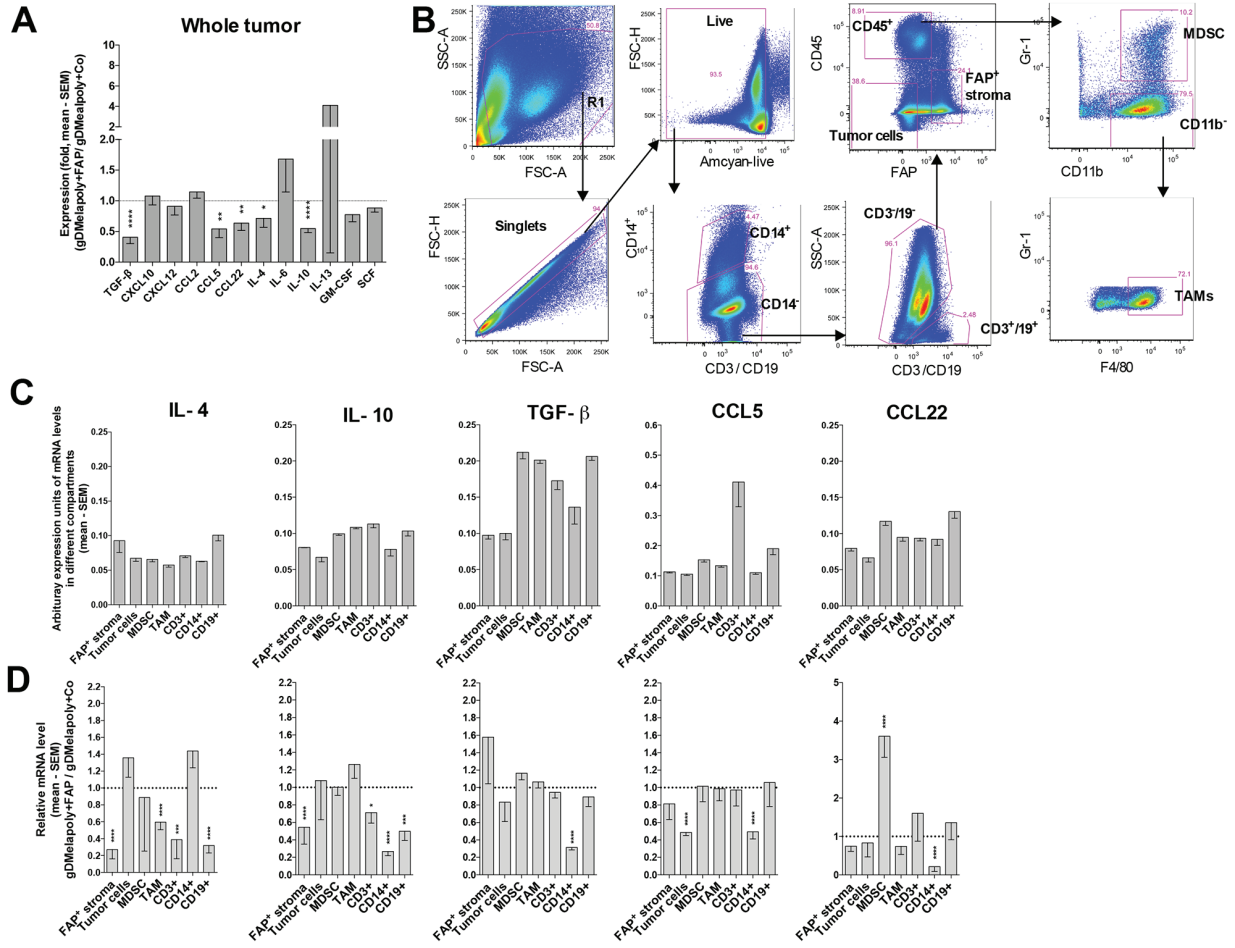
Depleting FAP^+^ stromal cells changes the TME's cytokine and chemokine profile Mice (n=7-8/group) were challenged with B16 tumors and vaccinated with AdC68-gDMelapoly mixed with control or AdC68-gDMelapoly mixed with AdC68-mFAP 3 days later. Tumors grown to about 1-1.5 cm in diameter were collected at necropsy (~4-5 weeks after tumor challenge). **A.** Relative mRNA expression levels of selected cytokines and chemokines in tumors from the AdC68-gDMelapoly+AdC68-mFAP group were compared to those from the AdC68-gDMelapoly+AdC69-gD control group. Data are shown as mean fold changes with SEM (TGF-β: p<0.0001; CCL5: p=0.0014; CCL22: p=0.0023; IL-4: p=0.030; IL-10: p<0.0001). **B.** Gating strategy used to sort different cell populations including FAP^+^ stromal cells, tumor cells, MDSCs, TAMs, CD3^+^ T cells, CD14^+^ cells and CD19^+^ B cells from B16 tumors. **C.** Abundance of selected cytokines and chemokines that show significantly reduced expression upon FAP^+^ stromal cell depletion in different cell populations of the TME. Tumor samples were from mice that received AdC68-gDMelapoly+control vector. Data of mRNA levels are shown as mean values of arbitrary expression units (1/(target gene Ct - GAPDH Ct)) with SEM. **D.** Relative mRNA levels of the indicated cytokines and chemokines in different cell populations from tumors of mice that received AdC68-gDMelapoly+AdC68-mFAP over those of mice that received AdC68-gDMelapoly+control vector (n=6/group). (IL-4: FAP^+^ stroma: p<0.0001, TAM: p<0.0001, CD3: p=0.000065, CD14: p=0.033, CD19: p<0.0001; IL-10: FAP^+^ stroma: p<0.0001, CD3: p=0.016, CD14: p<0.0001, CD19: p<0.0001; TGF-β: CD14: p<0.0001; CCL5: tumor cells: p<0.0001, CD14: p<0.0001; CCL22: MDSC: p<0.0001, CD14: p<0.0001).

We initially assessed whole tumors from mice that had been challenged 4-5 weeks before with B16 cells and received either AdC68-gDMelapoly with the control or the AdC68-mFAP vector three days later for a number of transcripts of cytokines and chemokine that may affect STAT signaling, ISCs recruitments or functions, or the balance of immune responses within the TME [35]. Upon including AdC68-mFAP into the vaccine regimen transcripts for CCL5, CCL22, IL-4, IL-10 and TGF-β significantly decreased (Figure 6A). Th2 related chemokines CCL5 and CCL22 preferentially recruit T cells that lack the capacity to eliminate tumor cells by direct lysis, i.e., regulatory T cells and Th2 cells; the latter bias immune responses away from Th1 [37]. Several cell subsets within tumors secrete IL-10 and TGF-β, which can contribute to immune suppression within the TME either through direct inhibition of cytolytic T cells or indirectly through the recruitment and activation of ISCs. TGF-β can further activate fibroblasts, which in turn promotes their immunosuppressive activities [28, 38]. IL-4 activates the STAT6 pathway in ISCs, which directly supports their suppressive functions. The reduction of these factors following AdC68-mFAP vaccination indicates a less immunosuppressive TME that reduces ISC recruitment and functions and supports CD8^+^T cell effector functions. Other factors, including C-X-C motif chemokine (CXCL) 10 and CXCL12, CCL2, IL-6 and IL-13, GM-CSF and stem cell factor (SCF) remained relatively stable upon addition of the AdC68-mFAP vaccine to AdC68-gDMelapoly.

To assess the origin of different factors, we sorted cells into tumors cells, FAP^+^ stroma cells, MDSCs, TAMs, and infiltrating leukocytes (T cells [CD3^+^], B cells [CD19^+^], macrophages and neutrophils [CD14^+^]) following the gating strategy shown in Figure 6B. Most factors originated from an array of different cell types (Figure 6C). FAP reduction decreased transcripts for IL-10, TGF-β, CCL5 and CCL22 from CD14^+^ cells (Figure 6D), of which 20-40% were F4/80^+^ TAMs; CCL5 transcripts were also reduced in tumor cells. Reductions in IL-4 and IL-10 transcripts were seen in numerous cell type including FAP^+^ stromal cells and inflammatory cells. The decreased transcripts of these cytokines/chemokines in different cell compartments within the TME suggests that reduction of FAP^+^ stroma cells has global effects that directly or indirectly affect other cell subsets. FAP-reduction did not affect cytokine or chemokine production by MDSCs and TAMs, indicating that their decreased pSTAT6 activation was caused by exogenous changes in cytokine levels. Collectively our data suggest that AdC68-mFAP vaccine changes the cytokine/chemokine milieu within the TME by reducing production of inflammatory factors. This may through down-regulation of STAT6 signaling pathways decreases the recruitment and immunosuppressive functions of ISCs.

### Reducing FAP^+^stromal cells reduces metabolic stress, decreases co-inhibitor expression and improves functions of vaccine-induced CD8^+^T cells

**Figure 7:**
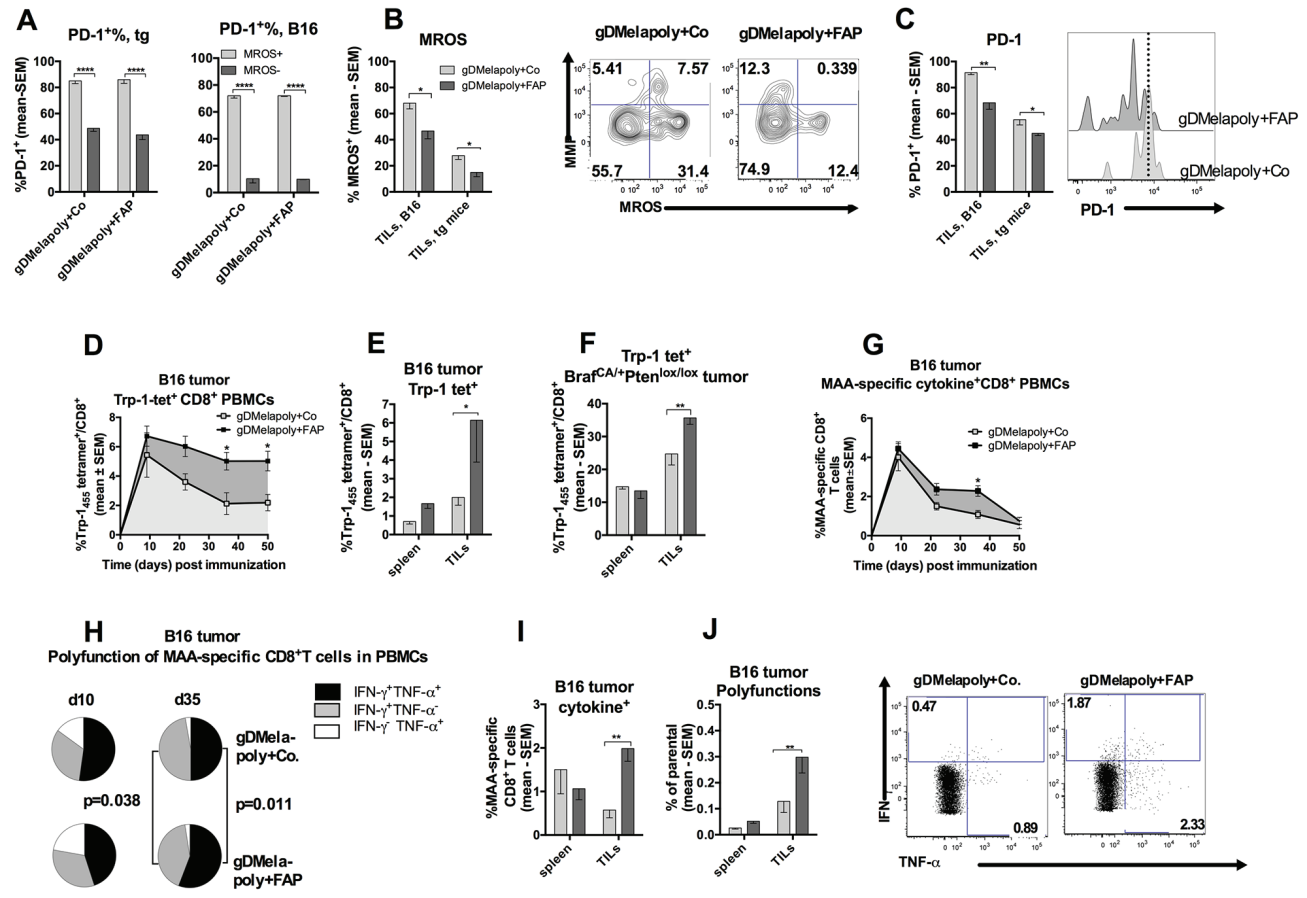
Depleting FAP^+^ stromal cells with AdC68-mFAP vaccine reduces metabolic stress and improves effector functions of AdC68-gDMelapoly-induced CD8^+^T cells **A.** Percentages of PD-1^hi^ cells within MROS^hi^ or MROS^lo^ Trp-1-specific CD8^+^TILs populations from tumors of mice that received either the control or the FAP vaccine. Both TILs from transgenic mouse tumors (left, n=5 mice/group) or C57/Bl6 tumors (right, n=5 mice/group) were analyzed. **B.** Percentages of Trp-1-specific CD8^+^T cells with high levels of MROS from similar sized transgenic tumors (collected 3 month after tumor challenge, n=4-5/group) or B16 tumors (collected 1 month after tumor challenge, n=9-14/group) of mice that received either AdC68-gDMelapoly+control (light grey) or AdC68-gDMelapoly+AdC68-mFAP (dark grey). TILs B16: p=0.018; TILs transgenic (tg): p=0.04. Flow plots show representative MMP and MROS expression in Trp-1^+^CD8^+^T cells from transgenic tumors of mice that received either AdC68-gDMelapoly+AdC68-gD or AdC68-gDMelapoly+AdC68-FAP. **C.** PD-1 expression on Trp-1-specific CD8^+^TILs from transgenic or B16 tumor-bearing mice that received either AdC68-gDMelapoly+AdC68-gDMelapoly (light grey) or AdC68-gDMelapoly+AdC68-mFAP (dark grey). Data are presented as percentages of Trp-1-specific CD8^+^T cells that show high expression of PD-1. TILs B16: p=0.0029; TILs transgenic (tg): p=0.045. Histograms: PD-1 expression on representative Trp-1-specific CD8^+^TILs samples from the transgenic tumors of mice that received either combination of vaccines. **D.** Percentages of Trp-1 tetramer^+^CD8+T cells in blood of mice (n=15/group) challenged with B16 tumor cells and vaccinated with either AdC68-gDMelapoly+AdC68-gD (empty square) or AdC68-gDMelapoly+AdC68-mFAP (black square). Responses were monitored for 50 days after vaccination. Responses compared by area under the curve (AUC): p=0.038; responses compared at individual time points on days 35: p=0.038; 42: p=0.047. **E.** Trp-1-specific CD8^+^T frequencies in spleens and tumors of B16-tumor bearing mice (n=9-14/group) that received AdC68-gDMelapoly+AdC68-gD (light grey bar) or AdC68-gDMelapoly+AdC68-mFAP (dark grey bar) were compared ~1 month after tumor challenge. Spleen: p=0.66, TILs: p=0.03. **F.** Trp-1-specific CD8^+^T frequencies in spleens and tumors of transgenic tumor-bearing mice (n=5/group) received AdC68-gDMelapoly+AdC68-gD (light grey bar) or AdC68-gDMelapoly+AdC68-mFAP (dark grey bar) were compared ~3 months after tumor induction. Spleen: p=0.70, TILs: p=0.0054. **G.** Mice (n=15/group) were challenged with B16 tumor cells and vaccinated with vectors three days later. Percentages of factor-producing CD8^+^T cells upon stimulation with the MAA-specific peptide pool was monitored in blood for 50 days after vaccination. Cells producing IFN-γ and/or TNF-α were measured and data are presented as the mean value of the sum of the responses with SEM. AUC: p=0.031; responses compared on day 35: p=0.032. **H.** MAA-specific CD8^+^T cell polyfunctionality in PBMCs of B16 tumor challenged mice from two vaccine groups were compared on days 10 and 35 after vaccination. Pie slice colors black: IFN-γ^+^TNF-α^+^, grey: IFN-γ^+^TNF-α^−^, white: IFN-γ^−^TNF-α^+^. **I.** Percentages of MAA-specific CD8^+^T cells from spleens and tumors of B16 tumor-bearing mice in each vaccine group that produced one or two cytokines at the time of necropsy (n=5/group). Light grey bar: AdC68-gDMelapoly+AdC68-gD, dark grey bar: AdC68-gDMelapoly+AdC68-mFAP. TILs p=0.006. **J.** Polyfunctions of MAA-specific CD8^+^T cells in spleen and tumors of B16 tumor-bearing mice in each vaccine group at the time of necropsy (n=5/group), data are presented as percentage of MAA-specific CD8^+^T cells that produce two cytokines. TILs p=0.0053. Flow blots show cytokines production in representative TIL samples from the different vaccine groups. Numbers on the corner indicate the percentages of CD8^+^T cells producing either IFN-γ or TNF-α.

It has been shown previously that FAP^+^ stromal cells suppress tumor-specific immune responses [23] and FAP^+^ cell depletion enhances tumor infiltration by T lymphocytes [21, 39]. We hypothesized that depleting FAP^+^ cells, which reduced the frequencies and functions of ISCs within the TME, may lessen metabolic stress and delay exhaustion of MAA-specific CD8^+^TILs as suggested by our *in vitro* co-culture assay. Indeed, increased metabolic stress indicated by high MROS levels was associated with enhanced expression of the co-inhibitor PD-1 on MAA-specific CD8^+^TILs from either transgenic or C57BL/6 mice (Figure 7A). In either transgenic or C57BL/6 mice with similar sized tumors, reducing FAP^+^ stromal cells significantly decreased percentages of MROS^hi^ Trp-1-specific CD8^+^TILs, especially those with lower mitochondrial membrane potential (MMP) (Figure 7B). Furthermore, co-inhibitor PD-1 levels were also significantly lower on MAA-specific CD8^+^TILs upon FAP^+^ cell depletion in both tumor models, suggesting that these cells were partially protected from exhaustion (Figure 7C).

To further determine whether destroying FAP^+^cells by AdC68-mFAP vaccination affects the functions of MAA-specific CD8^+^TILs, mice bearing 3 day-old B16 tumors were vaccinated with AdC68-gDMelapoly together with either the control or the AC68-mFAP vector. CD8^+^T cell responses to Trp-1_455-463_, the immunodominant epitope expressed by the AdC68-gDMelapoly vector, were monitored by tetramer staining of peripheral blood mononuclear cells (PBMCs) from the two vaccine groups over the course of 50 days. Depleting FAP^+^ tumor stromal cells significantly increased the overall Trp-1-specific CD8^+^T cell response in blood (p=0.038, area under the curve); differences were more pronounced at later time points, i.e. on days 35 and 50 after vaccination (Figure 7D). Mice were euthanized once tumors exceeded 1-1.5 cm in diameter and splenocytes and TILs were isolated. The mice that received the FAP vaccine exhibited significantly higher Trp-1-specific CD8^+^T cell responses in tumors (Figure 7E). The experiment was repeated with tumor-bearing Tyr::CreER, Braf^CA/+^Pten^lox+/lox+^ transgenic mice and results were comparable (Figure 7F). To assess whether the enhanced Trp-1-specific CD8^+^T cell frequencies were accompanied by an improvement of MAA-specific CD8^+^T cell effector functions, we tested PBMCs from B16 tumor-challenged mice for production of cytokines upon their *in vitro* stimulation with the MAA peptide pool, which included peptides representing the eight CD8^+^T cell epitopes expressed by the AdC68-gDMelapoly vaccine. Cells were then analyzed by intracellular cytokine staining (ICS) for production of IFN-γ and TNF-α. Overall frequencies of cytokine^+^CD8^+^T cells, i.e., the sum of percentages of T cells producing the two cytokines alone or in combination, were markedly higher in blood of mice that received the FAP vaccine together with the gDMelapoly vaccine (p=0.031, area under the curve) (Figure 7G). This effect was mainly observed at the later time point, i.e. on day 35 after vaccination. On day 10 after vaccination MAA-specific CD8^+^T cells from blood of the two vaccine groups showed comparable patterns for single or double functions; by day 35 T cells from mice that received the FAP vaccine were significantly more polyfunctional compared to those from the control group (Figure 7H). At the time of euthanasia mice that received both vaccines had higher percentages of factor-producing MAA-specific CD8^+^ TILs compared to those received AdC68-gDMelapoly with control vector (Figure 7I) and CD8^+^TILs were more polyfunctional (Figure 7J), i.e., a significantly higher percentage of MAA-specific CD8^+^TILs cells produced both IFN-γ and TNF-α.

Overall our data suggest that depleting FAP^+^ tumor stromal cells can decrease the metabolic stress of MAA-specific CD8^+^ TILs, which delay their differentiation towards functional exhaustion within the TME and result in significantly improved antitumor efficacy.

## DISCUSSION

Manipulating cells of the tumor stroma can achieve tumor regression. Decreases of MDSCs by factors that drive their differentiation towards mature antigen-presenting cells or macrophages has been shown to block their expansion or immune-inhibitory functions and prolong survival of tumor-bearing mice [40]. Reduced tumor progression is also achieved by targeting FAP, an antigen that is selectively expressed on fibroblasts present in tumors or at sites of chronic inflammation or wound healing [21-23, 41]. Mice with a genetic deletion of the FAP gene show prolonged survival after tumor challenge [42]. Accordingly, targeting FAP by active immunotherapy or T cells engineered to express a FAP-specific CAR decreases tumor growth and this is linked to reduced angiogenesis, changes in extracellular matrix proteins and augmented antitumor immunity [41]. Data presented here point to an additional mechanism in which an Ad vector designed to induce FAP^+^ cell-depleting CD8^+^T cells combined with a traditional cancer vaccine changes the immune balances within the TME by reducing levels of immunosuppression while enhancing functions of MAA-specific CD8^+^T cells through reducing their metabolic stress.

Our combination vaccine achieved complete remission of a transplantable highly aggressive B16 tumor in ~35% of mice while median survival of those that develop tumors was extended ~ 3 fold. This strategy also significantly prolonged survival of transgenic melanoma mice, although in this model the vaccine was given to mice, that already had substantial tumor burdens. It has been reported that eliminating FAP^+^ cells with CAR-T cells causes significant bone marrow toxicity and cachexia in some mice [14, 43], thus dampening enthusiasm for the use of FAP-targeting immunotherapy. We failed to witness significant adverse events in mice that received the FAP vaccine. These opposing results may reflect fundamental differences between vaccine-induced CD8^+^T cells, which recognize MHC class I-associated peptides, and CAR-T cells, which are triggered by cell surface expressed protein. Accordingly CAR-T cells have resulted in serious adverse events in human recipients due to off target activity against cells that express barely detectable amounts of the T cells' antigen [44]. Several other studies that tested FAP-specific CAR-T cells failed to observe significant toxicity in mice, which may reflect differences in the avidity and signaling capacity of different CARs [22, 23, 41, 45].

Combining the FAP vaccine with a TA-expressing vaccine resulted in increased T cell recruitment to tumors [45] and improved TA-specific CD8^+^T cell responses as has been reported previously [21, 39]. In our study numbers of MAA-specific CD8^+^T cells increased within tumors upon depletion of FAP^+^ cells. This was mainly due to less pronounced contraction of vaccine-induced CD8^+^T cells. Better preservation of vaccine-induced MAA-specific CD8^+^T cell response may also have been caused by the marked reduction of tumor-infiltrating ISCs after FAP^+^ stromal cell depletion.

MDSCs are a heterogeneous population of immature myeloid cells. In most cancers PMN-MDSCs, which are highly immune-suppressive, represent the majority of the total MDSC population. Targeted depletion of MDCSs increases survival of tumor-bearing mice [40, 46]. FAP^+^ fibroblasts through secretion of factors recruit and activate ISCs [37] and previous reports showed that an anti-fibrotic agent, which inhibits CAF functions, reduces ISC recruitment and functions [47, 48]. In our study, depletion of FAP^+^ cells upon vaccination reduced both MO- and PMN-MDSCs within the TME. This could reflect their reduced recruitment from the periphery, blockade of their expansion within tumors or increased differentiation into non immune-suppressive, more mature myeloid cells. Reduced expression of immune-inhibitors, such as iNOS and Arg1 by ISCs in FAP vaccine-treated mice argues for the latter mechanism.

STAT signaling plays a key role in fate decisions of ISCs. Specifically STAT3 controls their expansion, while STAT1 and STAT6 regulate activation of MDSCs and production of immune-inhibitory factors. The FAP vaccine reduced STAT6 signaling, which is the likely cause for the observed reduction of Arg1 and iNOS production by ISCs in FAP-vaccinated mice. STAT signaling in turn is driven by the surrounding cytokine and chemokine milieu that is maintained by different cells of the TME. In mice that received the FAP vaccine the profile of cytokine/chemokine transcripts present within tumors shifted with pronounced reductions in several of those known to activate STAT6 signaling. Further reductions were seen in transcripts of cytokines that promote Th2 at the expense of Th1 responses; the latter are typically associated with potent CD8^+^T cell responses. The membrane bound form of FAP reshapes extracellular matrix components, which in turn affects leukocyte/macrophages adhesion and migration. Reduction of FAP would thus be expected to remodel the composition of the tumor infiltrates and thereby the cytokine/chemokine milieu. Furthermore, factors secreted by FAP^+^ stromal cells can activate other cells of the TME, thus depleting FAP^+^ cells may affect the secretion of inflammatory factors by other cell populations. Indeed our data show that most of the factor-producing transcripts that changed upon FAP vaccination originated from tumor infiltrating leukocytes and CD14^+^ cells.

Elimination of FAP^+^ cells and the resulting reductions in numbers and functions of ISCs within tumors are associated with better preservation of MAA-specific CD8^+^TIL frequencies and improvement of their functions. In addition levels of MROS decreased in MAA-specific CD8^+^TILs accompanied by lower expression of the co-inhibitor PD-1. PD-1 signaling causes a gradual loss of CD8^+^T cell functions and eventually cell death [49, 50]. This is in part mediated by blockade of the Akt/mTOR pathway, which promotes glucose uptake, glycolysis and anabolic pathways. Within a hypoxic TME access to glycolysis may be especially crucial, as the alternative pathway of energy production, i.e., OXPHOS, requires O_2_. As has been described tumors commonly lack glucose due to its consumption by tumor cells [24] and presumably the tumor stroma. As remains to be investigated in more depth, it is feasible that depletion of FAP^+^ cells and reductions in ISCs affect metabolic pathways used by tumor cells and other cells within the TME and thereby increases the amount of glucose that is available to CD8^+^T cells. Access to glucose in turn would reduce T cell metabolic stress and decrease PD-1 expression [51], while promoting their proliferation and effector functions [24, 25]. We view decreased expression of PD-1 on vaccine-induced CD8^+^ TILs as a major benefit of FAP-vaccination, as blockade of PD-1 signaling by anti-PD-L1 or anti-PD-1 checkpoint inhibitors are showing remarkable success in delaying progression of fatal solid tumors in human patients [52, 53].

In summary, data presented here demonstrate that combining a traditional cancer vaccine with a vaccine that selectively targets FAP^+^ fibroblasts reduces tumor progression and even achieves cures in mouse melanoma models. Reduction of numbers and functions of tumor-infiltrating ISCs due to changes in the tumors' cytokine milieu and decreased STAT6 signaling in ISCs was identified as one of the underlying mechanism. Depletion of FAP^+^ cells and reductions in numbers and functions of ISCs lead to better preservation of vaccine-induced CD8^+^TIL functions. This is linked to decreased MROS and PD-1 levels signaling changes in the T cells' metabolism.

## MATERIALS AND METHODS

### Animal experiments

Female C57BL/6 mice (6-8 weeks) were purchased from the National Cancer Institute (NCI) and housed at the Wistar Institute Animal Facility. Tyr::CreER Braf^CA/+^ Pten^lox/lox^ transgenic mice were a generous gift from Dr. Xiaowei (George) Xu of the University of Pennsylvania (Philadelphia, PA). Experimental procedures were conducted following approved protocols. For C57BL/6 tumor challenge experiments B16Braf_V600E_ tumor cells were given subcutaneously (s.c.) into the right flank at 5×10^4^ cells/mouse. In transgenic mice, tumors were induced by applying 4-hydroxyltamoxifen (4-HT, Sigma, MO) to the shaved right flank at 4ug/mouse/day for three consecutive days. Tumor growth was monitored by measuring the perpendicular diameter of tumors every other day. Mice were euthanized once the diameter of tumor exceeded 1-1.5 cm. For combined vaccination experiments, AdC68-gDMelapoly was given at a dose of 10^10^ virus particles [vp] and AdC68-mFAP or AdC68gD vectors were given at the dose of 9×10^10^ vp. Vectors were diluted in phosphate buffered saline (PBS). For control groups, each mouse received 10^11^ vp of the AdC68gD vector. In single vaccination experiment, the AdC68-mFAP vector was given at 10^11^ vp per mouse. All vectors were given intramuscularly (i.m.). For T cell depletion assay, mice were challenged on day 0 with B16 tumor cells and vaccinated on day 3 with the AdC68-mFAP vector. Anti-CD8 (53-6.7) or anti-CD4 (GK1.5) or both antibodies (BioXCell, West Lebanon, NH) were given intraperitoneally at 0.3mg/mouse on day 0, 2 and 4 after tumor challenge.

### Cell lines

B16Braf_V600E_ cells (gift from Dr. M Herlyn, Wistar Institute), referred to as B16, were produced by transducing B16.F10 cells with lentiviral vector pLU-EF1a-mCherry expressing mouse Braf_V600E_. These cells rather than wild-type B16F10 cells were use as our vaccine carries the mutated Braf epitope. Ad vectors were grown in HEK 293 cells. Cells were grown in Dulbecco's Modified Eagles medium (DMEM) with 10% fetal bovine serum (FBS), and 1% penicillin-streptomycin.

### Ad vector production

The molecular clone of pAdC68-mFAP vector was constructed by digesting the pcDNA:mFAP vector (gift from Dr. E Puré, University of Pennsylvania) with ApaLI and ligating the mFAP insert into the pShuttle vector. The insert of mFAP from the pShuttle-mFAP vector was transferred to the molecular clone of AdC68 through I-CeuI, PI-SceI and PvuI digestion. Construction, rescue, purification, titration and quality control of AdC68 vectors have been described previously [27].

### Western blotting

HEK 293 cells were grown in 6-well plates until they reached 70-80% confluency. Medium was replaced with 1ml serum-free DMEM and different doses of Ad vectors from 10^9^-10^11^ vps were added to each well and incubated for two hours before 1ml of 10% FBS DMEM was added. AdC68-gD vector-transduced HEK 293 cells served as negative control. Cells were harvested 48 hours later, washed twice with cold PBS and lysed in RIPA buffer (Invitrogen, Grand Island, NY) with protease inhibitor (Roche, Indianapolis, IN). Protein samples were separated with 4-15% SDS-PAGE and transferred to a PVDF membrane. After blocking and washing, the membrane was incubated with primary sheep anti-FAP antibody (0.5ug/ml, R&D, Minneapolis, MN, AF3715) diluted with 5% milk and 0.1% Tween 20 in PBS overnight at 4°C. Secondary anti-sheep HRP antibody was used for protein detection. β-actin was probed as loading control as described before [54].

### Enzyme-linked immunosorbent assay (ELISA)

To measure FAP-specific antibody titers in AdC68-mFAP vaccinated C57BL/6 mice sera were collected in two-weekly intervals after vaccination. Briefly, ELISA plates were coated at 4°C overnight with mouse FAP (200ng/well, gift from Dr. J D Cheng, Fox Chase Cancer Center, Philadelphia, PA) diluted in coating buffer (0.1M NaHCO_3_, pH 9.6). Plates were washed with PBS/0.05% Tween- 20 and blocked using PBS with 10% BSA overnight. Serum samples were serially diluted in triplicates and incubated in wells for 2 hours at room temperature. Sheep anti-FAP antibody (Abcam, Cambridge, MA) was serially diluted as standard. After washing bound IgG was detected with alkaline phosphatase (AP) conjugated-goat anti-mouse secondary antibody for serum samples and AP-Donkey anti-sheep secondary antibody for the antibody standard (both from Abcam). A phosphatase substrate (Sigma, St. Louis, MO) dissolved in DEA buffer was added and absorbance was read about 20 minutes later at 405nm using an absorbance reader (ELx800, BioTek, Winooski, VT). Serum antibody titers were determined based on standard curves from each plate and are expressed as mg/ml.

### Tissue procession

Lymphocyte isolation from spleen and tumors has been described previously [27]. To prepare single cell suspensions, tumors were cut into <2mm small pieces and digested in 1mg/ml Collagenase/Dispase (Sigma) and 1mg/ml DNAse I (Roche) dissolved in Roswell Park Memorial Institute (RPMI) for 30-60mins on a shaker. 10mM EDTA was added after digestion, and single cells were prepared by mechanical mincing with metal-mesh sieves. Cells were then passed through a 70mm cell strainer.

### Antibody staining, flow cytometry and cell sorting

For intracellular cytokine staining, ~10^6^ lymphocytes were stimulated with peptides or peptide pools (5mg/ml/peptide) and Golgiplug (Fisher Scientific, Waltham, MA 1.5 μg/ml) dissolved in DMEM with 2%FBS for 5-6 hours at 37°C. (FAP peptides: FAP1: YSYTATYYI, FAP2: IQYLCWSPV, FAP3: LAYVYQNNI, FAP4: YVYQNNIYL, FAP5: SSWEYYASI, FAP6: RALTLKDIL, FAP7: YDLQNGEFV, FAP8: FAVNWITYL, FAP9: KALVNAQVD, FAP10: IAYSYYGDG, FAP11: TAVRKFIEM, FAP12: LTFWYKMIL, FAP13: SSDYYFSWL, FAP14: SQNHLYTHM, FAP15: IYSERFMGL, FAP16: HLYTHMTHF. MAA peptides: mTrp-1_455-463_: TAPDNLGYA, mTrp-1_481-489_: IAVVAALLL, mTrp-2_522-529_: YAEDYEEL, hTp-2_180-188_: SVYDFFVWL, hTrp-2_343-357_: STFSFRNAL, mTrp-2_363-371_: SQVMNLHNL, hgp100_25-33_: KVPRNQDWL, mBraf_594-602_: FGLANEKSI). A rabies virus glycoprotein peptide was used as negative control. After stimulation cells were stained as described previously [27, 55]. Cells were stained with Amcyan fluorescent reactive dye (Life technologies, Carlsbad, CA), anti-CD8-Alexa700 or –Brilliant violet (BV) 605 and CD44-FITC or -PercpCy5.5. For intracellular cytokine staining cells were stained with antibodies to IFN-γ(APC or BV421), TNF-α (PE-Cy7, Biolegend, San Diego, California), granzyme B (APC, Life Technologies) and perforin (PE, eBioscience, San Diego, CA) as described [54]. For Trp-1_455_ tetramer staining, cells were stained with PE-labeled Trp-1-specific MHC class I (H-2D^b^) tetramer with TAPDNLGYM peptide (NIAID tetramer facility, Atlanta, GA) together with other surface markers including anti-CD8, CD44, and PD-1-BV605 (all from Biolegend, San Diego, CA). For mitochondrial membrane potential (MMP) and mitochondrial reactive oxygen species (MROS) staining, cells were stained with DioC_6_ (40nM) and Mitosox red (5 μM, Life technologies) for 30mins at 37°C before surface markers staining. For staining of other cell populations from tumors, single cell suspensions were blocked with CD16/CD32 Fc receptor blocking antibody (BD Pharmingen, San Jose, CA) for 30 mins at 4°C. Cells were further stained with either sheep anti-FAP antibody (10μg/ml, R&D, AF3715) or normal sheep IgG control antibody (R&D) at 4°C for 1 hour. After washing cells were stained with donkey anti-sheep APC-conjugated secondary antibody for 30 minutes together with anti-CD3-Pacblue, CD14-PercpCy5.5, CD19-FITC, Gr-1-PE, CD11b-PE-Cy7, CD206-BV605, F4/80-Alexa700, Sca-1-PE-Cy7 and CD90.2-FITC (all from Biolegend). For staining of immunosuppressive functions tumor-derived cells were first stained with CellRox green (5mm) for 30 mins at 37°C. After washing, cells were stained with surface markers for 30 mins at 4°C. Cells were then fixed and permeabilized with fixation/permeabilization buffer (Becton Dickinson, Franklin Lanes, NJ) for 30 mins on ice, followed by staining with rabbit polyclonal antibody to iNOS (10mg/ml, Abcam) in 1x permwash buffer (Becton Dickinson) for 30mins on ice. Normal rabbit IgG (R&D) was used as isotype control. Following washing cells were further stained with Alexa647-goat anti rabbit secondary antibody (1:2000, Life Technologies) and Arginase1 PE (R&D) for 30 min at 4°C. To measure expression of phosphorylated STAT, cells were first fixed with pre-warmed Fix Buffer I (pre-warmed to37°C, Fisher) for 10 mins at 37°C. After washing with cell staining buffer (Biolegend), cells were stained with surface markers and permeabilized with PermBuffer III (pre-chilled to −20°C, Fisher) for 30 min at 4°C. Cells were washed twice with cell staining buffer and stained with STAT1(pY701) PercpCy5.5, STAT3(pS727) Alexa647 and STAT6(pY641) antibodies (all from Fisher) diluted in cell staining buffer for 60mins at room temperature. Cells were analyzed by an LSRII (BD Bioscience). Data were analyzed with FlowJo (TreeStar, Ashland, OR).

### *In vivo* cell lysis assay

C57BL/6 mice were vaccinated with either AdC68-gD or AdC68-mFAP vectors. Two weeks later splenocytes from naïve syngeneic mice were plated at 10^7^ cells/100ul and pulsed with FAP peptides 1,5,7,8,9 (these peptides represents FAP-derived CD8^+^T cell epitopes with high immunogenicity in C57/Bl6 mice), or a control peptide from the rabies glycoprotein at 5mg/ml for each peptide at 37°C for 2 hours. Following washing cells pulsed with FAP peptides were labeled with carboxyfluorescein succinimidyl ester (CFSE, Life technologies) at 2mM, while cells pulsed with the control peptide were labeled with CFSE at 0.2mM. Two cell populations were mixed at 1:1 ratio and a total of 2×10^7^ cells were transferred into mice vaccinated with either vector through tail vein injection. 16 hours later, splenocytes were isolated from recipient mice and live single cells were analyzed by flow cytometry for expression of CFSE. Loss of CFSE^hi^ cells pulsed with FAP peptides was used as a measure of specific lysis. Percentage of cell lysis was calculated using the following formula: (1-(%CFSE^lo^ cells in control vaccinated mice/%CFSE^hi^ cells in control vaccinated mice)/(%CFSE^lo^ cells in FAP vaccinated mice/%CFSE^hi^ cells in FAP vaccinated mice)) x100

### Gene expression analysis

For the analyses of transcripts from whole tumors, mice were perfused immediately after euthanasia with PBS and heparin (10 units/ml). The tumors were cut into small pieces, stabilized with RNA *later* RNA stabilization reagent (Qiagen, Valencia, CA) and stored at −80°C until processed for RNA isolation. To analyze transcripts in different tumor cell subsets, single cell suspensions were prepared and stained as described above. Cells were sorted (Mono Astrios, Beckman Coulter, Jersey City, NJ) on ice into RNAprotect cell reagent (Qiagen). RNA was isolated using RNeasy plus mini kit (Qiagen) and RNA concentration was determined by Nanodrop (Thermo Scientific, Waltham, MA). Reverse transcription was performed using the high capacity cDNA reverse transcription kit (Life Technologies) and relative quantitative real-time PCR was performed using Fast SYBR Green master mix and 7500 Fast Real-time PCR system (Life Technologies). All primers (listed below) were designed by Vector NTI. GAPDH is used as the internal control. The following primers were used (forward followed by reverse): CCL2: 5′-TGCTGACCCCAAGAAGAAATG-3′, 5′-TGAAGAC CTTAGGGCAGATGCAG-3′; CCL5: 5′-AGCTGCCCTC ACCATCCTC-3′, 5′-AGCGCGAGGGAGAGGTAGG-3′; CCL22: 5′-ACTCCTGGTGGCTCTCGTCC-3′, 5′-TGG CAGAGGGTGACGGATGTA-3′; CXCL10: 5′-AAGGAC GGTCCGCTGCAAC-3′, 5′-TGATCTCAACACGTGGG CAGG-3′; CXCL12: 5′-TCGCCAGAGCCAACGTC AAG-3′, 5′-TCGGGTCAATGCACACTTGTCTG-3′; IL-4: 5′-AACCCCCAGCATGTTGTCATCC-3′, 5′-TGGCGT CCCTTCTCCTGTGAC-3′; IL-6: 5′-ACAAAGCCAG AGTCCTTCAGAGAG-3′, 5′TTGGAAATTGGGGTAG GAAGG-3′; IL-10: 5′-AAGGTGTCTACAAGGCCATG AATG-3′, 5′-TGTCTAGGTCCTGGAGTCCAGC-3′; IL-13: 5′-TGCTTGCCTTGGTGGTCTCG-3′, 5′-TGCCG TTGCACAGGGGAGTC-3′; TGF-β: 5′-TACGTCAGAC ATTCGGGAAGC-3′, 5′-TTCAGCCACTGCCGTACAA C-3′; GM-CSF: 5′-ACCCACCCGCTCACCCATC-3′, 5′-TCTTCAGGCGGGTCTGCACAC-3′; SCF: 5′-ACC AAGGAGATCTGCGGGAATC-3′, 5′-ACATCCATCCC GGCGACATAG-3′, GAPDH: 5′-TGCCCCCATGTT TGTGATGG-3′, 5′-AATGCCAAAGTTGTCATGGATGACC-3′.

### MDSC *in vitro* co-culture assay

Gr-1^hi^CD11b^+^MO-MDSCs, Gr-1^int^CD11b^+^PMN-MDSCs and CD206^+^F4/80^+^ TAMs from spleens of mice bearing 1 month-old Braf^CA/+^Pten^lox/lox^transgenic tumors or B16 tumors were sorted into RPMI medium. CD8^+^T cells were purified from spleens of naive C57BL/6 mice by negative selection using magnetic beads (MACS, Stemcell Technologies, Vanc ouver, Canada). For inhibition assays, following isolation CD8^+^T cells were labeled with celltrace violet dye (1μM, Life Technologies, Carlsbad, CA) at 37°C for 20 min. MDSCs or TAMs (8×10^4^ cells/well) and CD8^+^T cells (4×10^5^ cells/well) were plated at a 1:5 ratio into wells of a 96-well plate pre-coated with anti-CD3 antibody (5μg/ml, 4°C overnight, BD Bioscience, Minneapolis, MN) in RPMI medium (Life Technologies) supplemented with 10% FBS (Life Technologies), 20mM HEPES, 2mM Glutamax, 1mM sodium pyruvate, 0.05mM 2-mercaptoethanol and 1% penicillin-streptomycin. Anti-CD28 antibody (1μg/ml, BD Bioscience) and mouse IL-2 (20U/ml, Roche) were added to each well. Stimulated CD8^+^T cell without MDSCs/TAMs served as positive controls, while CD8^+^T cells cultured without activators were used as negative controls. MROS and PD-1 levels on T cells as well as T cell proliferation were analyzed on day 5 of culture by antibody staining and flow cytometry. T cell proliferation data are shown as Proliferation index (PI), i.e., the average number of divisions using the formula:

$$PI=\frac{\sum_{i=2}^{N-1}\frac{n_{i}}{2^{i-1}}\times \left(i-1\right)}{\sum_{i=2}^{N-1}\frac{n_{i}}{2^{i-1}}}$$

*n*_*i*_ is the cell number of the *i*-the generation (*i*=1,2,3,…, *N*).

### Statistical analyses

Significance of differences between 2 populations was calculated by Student's t test; significance of differences among multiple populations was calculated by one-way or two-way ANOVA using GraphPad Prism 6. Type I errors were corrected for multiple comparisons using the Holm-Sidak method. Overall responses over time were calculated by area under the curve (AUC) analysis for each animal followed by student's t test comparing AUC values. Differences in survival were calculated by Log-rank Mantel-Cox test. Significance was set at p-values of or below 0.05.
